# Effects of media use, smart phone addiction, and adult ADHD symptoms on wellbeing of college students during the COVID-19 lockdown: Dispositional hope as a protective factor

**DOI:** 10.3389/fpsyg.2022.1019976

**Published:** 2022-12-23

**Authors:** Roxana Andreea Toma, Craig A. Anderson, Marius Matichescu, Anca Franţ, Bogdan Almǎjan-Guţă, Adela Cândea, Kira Bailey

**Affiliations:** ^1^Department of Psychology, West University of Timişoara, Timişoara, Romania; ^2^Department of Psychology, Iowa State University, Ames, IA, United States; ^3^Department of Sociology, West University of Timişoara, Timişoara, Romania; ^4^Teacher Training Department, West University of Timişoara, Timişoara, Romania; ^5^Department of Kinetotheraphy and Special Motricity, West University of Timişoara, Timişoara, Romania; ^6^Department of Psychology, Ohio Wesleyan University, Delaware, OH, United States

**Keywords:** hope, ADHD, wellbeing, smart phone addiction, college students

## Abstract

**Introduction:**

The present study investigated the role of dispositional hope as a potential protective factor moderator in the relationship between adult ADHD symptoms, media use/smart phone addiction and wellbeing during the period of isolation because of the COVID-19 pandemic among students in Romania.

**Methods:**

A sample of 333 college students (86.8% female and 13.2% male) between the age of 18 and 47 with a mean of 20.6 years old from West University of Timişoara completed online surveys. Mediation and moderation analyses were performed to assess the associations among the variables.

**Results:**

Results confirmed the negative associations of both adult ADHD and smartphone addiction with overall wellbeing. The smartphone addiction/ wellbeing association was moderated by dispositional hopefulness, such that high hopefulness served as a protective factor [*b* = −0.008, 95% percentile CI (−0.0134; −0.0012)].

**Discussion:**

Implications for the educational environment are discussed.

## 1. Introduction

With the COVID-19 outbreak, students have faced unprecedented challenges in adapting to distance learning and spending most of their daily activities online on a smartphone or computer. It is well-recognized that the use of informatics and communication technologies presents both opportunities and risks (Arrivillaga et al., 2020; Plante et al., 2020; Stavropoulos et al., 2021). Prior studies showed that problematic media use and phone dependency can lead even to more problematic cognitions like suicidal thoughts (Arrivillaga et al., 2020) or emotional problems like anxiety for those who had low exposure to media before the pandemic (Magson et al., 2021). Furthermore, there are gender differences in problematic social media use; for instance, it becomes an independent risk factor for negative health behaviors of adolescents, especially girls (Buda et al., 2021). One of the aspects that experts on media and internet use agree upon is that individual differences in media use result in significant media effects on the user, some of which are harmful (Anderson C. A. et al., 2017; Arrivillaga et al., 2020; Stavropoulos et al., 2021). For this reason, it is important to explore potential protective factors that might reduce the harmful effects of internet (and smartphone) use.

In Romania, physical isolation caused by the quarantine period during the COVID-19 pandemic led students to increase their daily activities in online environments (e.g., classes). The students also spent significant time at night online (Buda et al., 2021). Essentially, online environments became the gateway to all forms of knowledge and leisure. Students moved from classrooms to online classes using computers, smartphones, and/or tablets. Similarly, the COVID-19 pandemic lockdown in the spring of 2020 prohibited many face-to-face leisure activities, increasing internet use for leisure and non-academic pursuits.

Worldwide researchers made efforts to depict the realities and evaluate the impact of the COVID-19 pandemic and its effects at the psychological level. For adolescents and young adults, one of the main concerns was related to psychological wellbeing. For example, results showed that young people reported poor outcomes in mental and psychological wellbeing when living under COVID-19 pandemic restrictions (Zolopa et al., 2022). In Romania, most public institutions were closed, including Romanian Universities. Classes and related university activities were moved online. Research on students' perceptions of and coping strategies used during the lockdown revealed not only that students reported more emotional issues (Waselewski et al., 2020) but also that they used coping strategies with positive outcomes like maintaining positivity and staying connected (Waselewski et al., 2020; Magson et al., 2021).

Although only 3 years have passed since the COVID-19 pandemic emerged and spread worldwide, some moderators for lockdown effects on psychological wellbeing have already been identified, such as emotional intelligence (Arrivillaga et al., 2020), gender (Magson et al., 2021; Sürücü et al., 2021), conflict with parents, COVID-19 distress, social disconnection, stay home adherence (Magson et al., 2021) and hope (Christens et al., 2013; Demirli et al., 2015; Stoyles et al., 2015; Li et al., 2021).

Our research team took a different approach to these pandemic-based problems. Specifically, we investigated dispositional hope as a potential moderating protective factor in the links between adult ADHD symptoms,^1^ smartphone addiction, and psychological wellbeing. Our main research question was whether, in times of physical isolation and higher online exposure and communication, hopefulness can positively influence students' wellbeing by reducing the known harmful links between self-reported adult attention deficit problems and addiction to smartphones. We chose to investigate the role of hope as a protective factor for students' wellbeing for two reasons, one based on theory and the other based on recent research on students' performance during the COVID-19 pandemic times. Theoretically, students' hope is investigated as one of the dimensions of psychological capital (PsyCap) (Luthans and Youssef, 2004; Luthans et al., 2007). Although a concept mainly studied in organizational psychology, the dimensions of PsyCap have been investigated as resources for wellbeing related to students' behavior during the COVID-19 pandemic. Studies showed that students needed coping strategies for meeting their needs during the COVID-19 pandemic (Griggs, 2017; Waselewski et al., 2020) and that hope, as a personal resource, enhanced student's wellbeing (Griggs, 2017; Putrawan et al., 2021). We explored if students' hope might have served as a protective factor in their general wellbeing during the pandemic, as the main effect (e.g., hope positively associated with wellbeing) and as an interactive moderator (e.g., reducing the negative association between ADHD symptoms and wellbeing). Recent research reveals worse psychological wellbeing during the isolation period of the COVID-19 pandemic for multiple age groups, from children and adolescents (Grey et al., 2020; Vallejo-Slocker et al., 2020; Waselewski et al., 2020; Magson et al., 2021) to college students (Savage et al., 2020; Evans et al., 2021; Thorisdottir et al., 2021). A similar protective effect also appeared for the people living in the city and apartments as opposed to those who had a green space (Mastorci et al., 2021). These findings led to the conclusion that further monitoring of the wellbeing of children, adolescents, and young people was needed (Vallejo-Slocker et al., 2020).

In a rapid review of 21 studies investigating psychological wellbeing during the COVID-19 pandemic, Zolopa et al. (2022) found that all the studies showed a decrease in psychological and mental wellbeing for children, adolescents, and young people. The same study also reviewed eight mainly qualitative studies that focused on resilience and coping strategies for adapting to the pandemic situation and lockdown. They demonstrated the supportive role of resilience and coping strategies - like staying connected, maintaining positivity, exercising, engaging in creative activities, or adopting problem-solving focus (Branquinho et al., 2020; Waselewski et al., 2020; Scott et al., 2021). Positive coping strategies included staying connected, maintaining positivity (Waselewski et al., 2020), establishing a routine, and carrying out pleasurable activities (Branquinho et al., 2020). Among the resilience and coping strategies that helped young adults adapt to the situation of lockdown, Griggs (2017) identified 20 quantitative studies that focused on the role of hope in enhancing the wellbeing of students.

### 1.1. Hope as a psychological resource

Dispositional (trait) hope is considered an important factor in general wellbeing (Redlich-Amirav et al., 2018). An integrated review of 20 quantitative studies exploring hope and mental health in young adult college students concluded that dispositional hope appears to be a protective moderator between depression and negative life events, and a protective factor in suicide and healthy behavior engagement (Griggs, 2017). We embraced the conceptual framework of Snyder's theory on adult hope and chose Snyder's Adult Hope Scale (Snyder et al., 1991) as a measure of hope for two reasons. The first was related to the conceptual construction of hope as a unidimensional concept derived from the cognitive-behavioral approach related to personal goals and one of the main dimensions of the PsyCap. The second reason was the psychometric properties of the scale (Redlich-Amirav et al., 2018); the hope scale is a trait-like measure of hope (Feldman et al., 2009). Snyder's *Hope* theory views hope as a motivational trait that strengthens the individual's self in pursuing his or her relevant goals. Furthermore, hope adjustment for individuals is related to the way they experience success or failure in pursuing their goals (Feldman et al., 2009).

The reasons that we chose to test the moderating role of hope as a protective factor during the lockdown caused by the COVID-19 pandemic were the following: (a) hope proved to be a moderator in increasing wellbeing in relation to negative life events (Hirsch et al., 2012; Christens et al., 2013; Visser et al., 2013; Sun et al., 2014; Hellman and Gwinn, 2017; Munoz et al., 2020; Li et al., 2021; Sparks et al., 2021), (b) the moderating effect of hope appears unaffected by ethnicity (Hirsch et al., 2012; Visser et al., 2013), and (c) its moderating effect showed stability over time—at least at two years' difference (Marques et al., 2011).

To further test the possible moderating role of hope and lockdown-related maladaptive behaviors, we searched for problematic behavior related to heavy online exposure and settled on problematic internet use through computers/smartphones/tablets.

### 1.2. Media use and smartphone addiction

Problematic internet use is broadly defined as an individual's inability to control his or her use of the internet, spending excessive amounts of time online, and leading them to distress and /or impairment in their everyday life (Anderson E. L. et al., 2017). There is a debate on the degree of overlap among the construct of problematic internet/media use and problematic smartphone use and whether researchers should consider the latter a subcategory of internet use or a separate construct (Cheever et al., 2018). In the present research, we assessed media use and problematic smartphone use separately, the latter conceived in terms of addiction. Even before the pandemic, problematic smartphone use was significantly associated with suicide ideation in Spanish adolescents (Arrivillaga et al., 2020). The frequent use of devices to connect to the internet has also been linked to psychological wellbeing. For example, Girela-Serrano et al. (2022) concluded that high bedtime use of phones or mobile devices was associated with lower wellbeing, whereas moderate use may improve psychological wellbeing by strengthening social connections and providing support. On the other hand, problematic smartphone use leads to behaviors that are conceptualized in a way that is very similar to behavioral addiction (Girela-Serrano et al., 2022). This approach defines problematic smartphone use as addiction and includes loss of control (trouble limiting smartphone use), tolerance (needing more smartphone use to achieve the same psychological rewards), and withdrawal (Harris et al., 2020).

Numerous studies found negative effects of excessive or problematic use of the internet on young people (Buda et al., 2021) and on young people's psychological states such as self-esteem (Midgley et al., 2021), wellbeing (Vanden Abeele et al., 2022), mental health (Girela-Serrano et al., 2022), attention and aggression (Swing and Anderson, 2014), and other behaviors (Girela-Serrano et al., 2022). More broadly, problematic internet use is viewed as a type of addiction (e.g., Brand et al., 2016; Bender et al., 2020).

Similarly, Zolopa et al.'s (2022) rapid review of the studies that investigated psychological wellbeing during the COVID-19 pandemic found that there were changes in the externalizing behavior of adolescents and young people with ADHD symptoms. For example, a longitudinal study of adolescents with or without ADHD showed significant increases in inattention but no change in hyperactivity (Breaux et al., 2021; Zolopa et al., 2022). These longitudinal findings showed an increase in inattention among individuals when highly technologically stimulated, even in adolescents without prior ADHD symptoms, and therefore the need for additional research on protective psychological factors (Breaux et al., 2021). A study on Italian children and adolescents with ADHD during lockdown due to the COVID-19 pandemic reported the participants' different mood patterns according to the severity of the ADHD. Those with a moderate and severe degree of ADHD showed an improvement in emotional mood and behavioral dimension, and the ones with a low-severity degree of ADHD showed increases in boredom, temper tantrums, argumentativeness, and aggression (Melegari et al., 2021).

In sum, prior research suggests that the negative association between ADHD symptoms and wellbeing may be at least partially mediated by smartphone addiction and that dispositional hope may act as a moderator.^2^

### 1.3. The present study

In this context, we examined the way online media seized the daily life of Romanian college students, how they perceived their state of wellbeing, and if hope served a protective function. Our main research goal was to investigate the potential protective role of hope on the effects of a highly technologically stimulated environment on wellbeing, both as a main effect *and* as a moderator (interactive effect). More specifically, we predicted: (a) a positive association between adult ADHD symptoms and smartphone addiction; (b) a negative association between ADHD symptoms/smartphone addiction and psychological wellbeing; and (c) a positive association between hope and wellbeing. We also tested: (d) potential moderating effects of dispositional hope on the association between ADHD symptoms and psychological wellbeing and on the association between smartphone addiction and wellbeing, such that those with high dispositional hope would show fewer associations between ADHD symptoms and smartphone addiction on wellbeing than would those with low dispositional hope.

## 2. Materials and methods

We conducted an online self-report survey to investigate the situation of Romanian students attending different specializations at WUT University, all of whom had their originally face-to-face classes moved online during the beginning of the pandemic. Data collection occurred 2 months after the lockdown in May 2020.

The online survey was programmed with QuestionPro and consisted of demographic data and five measurements of the main variables—adult ADHD, smartphone addiction, media use, dispositional hope, and wellbeing.

For measuring media use, we utilized a few items from the *General Media Habits Questionnaire - Adult Version (modified)* (Gentile et al., 2009). The items assessed total online times per week and combined into a media use score. We asked participants to respond two times to these items, first with reference to time spent on devices before the pandemic and the second with reference to time spent during the lockdown. This allowed us to investigate retrospectively on-screen time at two moments of the pandemic—before the lockdown and 2 months after isolation.

For investigating ADHD symptoms, we used the Adult ADHD Self-Report Scale (ASRS), Kessler et al. (2005) developed in cooperation with the World Health Organization. It assessed respondents' attention deficit symptoms present in the past 6 months. The screening tool consists of 18 questions and assesses responses on a scale of five points *never, rarely, sometimes, often*, and *very often*. Adult ADHD has two subscales—inattention and hyperactivity-impulsivity. Six of the 18 questions constitute the ASRS screener and seven of the questions are considered clinically significant symptom levels. We chose ASRS because it spots inattention symptoms as well as clinical conditions.

The Smartphone Addiction Scale—short version (SAS-SV; α = 0.911) (Kwon et al., 2013) was administered to measure addictive behaviors related to smartphone use. It has 10 self-report items with responses on a six-point scale (1: strongly disagree to 6: strongly agree). It assessed three symptoms, addiction, tolerance, and withdrawal, with the cutoff points of 31 (sensitivity of 0.86 and specificity of 0.89) for boys and 33 (sensitivity of 0.87 and specificity of 0.88) for girls.

We assessed psychological wellbeing with the 30-item self-report Mental, Physical and Spiritual Well-Being scale (MPS) (Vella-Brodrick and Allen, 1995). Three subscales (10 items each) assess behaviors in mental, physical, and spiritual wellbeing with a five-point frequency format (*often* to *never*).

Finally, we chose Adult Hope Scale (AHS) (Snyder, 1994) for measuring dispositional hope. It has 12 self-report items answered on an eight-point scale (*definitely false* to *definitely true*) to measure the respondent's level of hope. The scale is divided into two subscales—agency and pathways—each of them consisting of four items, the remaining four items are fillers.

The main goals of the study were to investigate if media use, smartphone addiction, and attention problems became more problematic during the lockdown, if these variables were associated with wellbeing for Romanian students studying online during the COVID-19 pandemic lockdown, and if dispositional hope can serve as a protective factor for enhancing students' wellbeing, taking into consideration the particular situation of the lockdown learning environment.

### 2.1. Participants

The participants were 333 college students aged between 18 and 47 (*m* = 20.66, SD = 4.8), 86.8% women and 13.2% men from WUT courses specializing in psychology, sociology, languages, and sports (physical education and kineto-therapy).

### 2.2. Statistical analysis

The statistical analyses were conducted in SPSS. We excluded item seven from the smartphone addiction scale because of its low value in the reliability analysis. Also, we did not include a separate scale in the analysis of mental wellbeing as with a value of 0.58 it did not show a reliable internal consistency as a separate sub-scale.

We expected to find an increase in media use (*via* smartphones/tablets/computers) during the pandemic. We used mean differences to test pre- and post-lockdown media use. We also expected to find a negative association between wellbeing and both adult ADHD symptoms and smartphone addiction and a positive association between hope and wellbeing. Zero-order correlations and multiple regression analyses were used to test these hypotheses. Most importantly, we investigated whether hope had a protective influence by conducting a moderated mediation analysis.

## 3. Results

As can be seen in Table 1, all variables met the basic assumptions for performing the planned regression and moderation analyses.

**Table 1:** Descriptive statistics.

**Variable**	***M***	***SD***	**Min**	**Max**	**Skewness**	**Kurtosis**
Adult hope scale_agency	24.80	5.06	4	32	−0.96	1.07
Adult hope scale pathways	25.79	4.87	5	32	−1.24	2.12
Adult hope scale _total	50.6	9.34	9	64	−1.13	1.75
ADHD_adult	25.77	10.98	0	59	0.148	0.011
Smartphone addiction	30.6	12.57	10	60	0.215	−0.813
Wellbeing_physical	30.2	6.2	12	50	−0.110	0.223
Wellbeing spiritual	34.54	7.44	15	50	−0.032	−0.555
Wellbeing total	99.95	11.74	68	143	0.160	0.146

N = 333.

### 3.1. Pre- vs. post-lockdown media use

As expected, there was an increase in media use from before the pandemic (*m*1 = 13.66, SD = 20.84) compared to the reported media use during the first pandemic lockdown (*m*_2_ = 17.25, SD = 22.66). A dependent *t*-test showed a small but significant increase *t*_(332)_ = 3.44, *p* ≤ 0.001 with a Cohen *d* = 0.37.

### 3.2. Gender differences in hope and wellbeing

Because problematic internet and media use often differ by gender, we tested gender effects on the hope and wellbeing measures. The sample included 44 men and 289 women. The results revealed that the male and female participants differed in their levels of hope and physical and spiritual wellbeing (see Table 2).

**Table 2:** Descriptive statistics for variables hope and wellbeing related to gender.

**Sex**	***N***	**Hope agency**		**Hope pathways**		**Adult hope total**		**Wellbeing_physic**		**Wellbeing_spiritual**	
		**Mean**	**SD**	**Mean**	**SD**	**Mean**	**SD**	**Mean**	**SD**	**Mean**	**SD**
Men	44	23.34	6.22	24.22	5.49	47.56	11.1	32.75	5.46	32.4	8.34
Women	289	25.03	4.84	26.03	4.74	51.06	8.97	29.82	6.23	34.87	7.26

Women scored higher than men on total dispositional hope, *t*_(331)_ = 2.33, *p* < 0.05, Cohen *d* = 0.38, as well as on both subscales — hope agency [*t*_(331)_ = 2.07, *p* < 0.05, *d* = 0.34] and hope pathways [*t*_(331)_ = 2.31, *d* = 0.37].

There was no gender difference in the general level of wellbeing of the participants. However, women scored lower than men on physical wellbeing, *t*_(331)_ = −2.95, *p* < 0.05, Cohen *d* = −0.48. Conversely, women scored higher than men on spiritual wellbeing, *t*_(331)_ = 2.06, *p* < 0.05, two-tailed, Cohen *d* = 0.33.

### 3.3. Zero-order correlations

Table 3 reports the correlations among the key variables. As predicted, dispositional hope (Hope_Tot) was significantly and positively related to physical (*r* = 0.243, *p* < 0.01), spiritual (*r* = 0.342, *p* < 0.01), and overall wellbeing (*r* = 0.495, *p* < 0.01). Also note that both hope subscales were positively associated with all three wellbeing subscales.

**Table 3:** Correlation matrix and results of reliability analysis.

**Nr**	**Variable/*R*/**	**1**	**2**	**3**	**4**	**5**	**6**	**7**	**8**	**9**	**10**
1	Hope_agency	0.81									
2	Hope_pathways	0.765^**^	0.82								
3	Adult hope total	0.942^**^	0.937^**^	0.89							
4	Adult ADHD	−0.294^**^	−0.206^**^	−0.267^**^	0.88						
5	Smartphone addiction	−0.222^**^	−0.230^**^	−0.240^**^	0.503^**^	0.90					
6	Wellbeing physic	0.246^**^	0.211^**^	0.243^**^	−0.439^**^	−0.339^**^	0.68				
7	Wellbeing spiritual	0.387^**^	0.252^**^	0.342^**^	0.012	−0.056	−0.099	0.76			
8	Wellbeing total	0.533^**^	0.395^**^	0.495^**^	−0.316^**^	−0.304^**^	0.456^**^	0.731^**^	0.68		
9	Media use pandemic time	−0.080	−0.045	−0.067	0.086	0.076	−0.086	0.005	−0.072	–	
10	Media use before pandemic	−0.057	−0.008	−0.035	0.162^**^	0.118^*^	−0.038	−0.015	−0.056	0.621^**^	–

N = 333.

** p < 0.01.

* p < 0.05.

Diagonal values are internal reliabilities for multi-item scales.

Also as expected, dispositional hope yielded significant negative correlations with adult ADHD symptoms (*r* = −0.267, *p* < 0.01) and smartphone addiction (*r* = −0.240, *p* < 0.01). Note that these were non-significantly smaller than the hope/wellbeing correlations. Furthermore, we found a significant positive correlation between ADHD symptoms and smartphone addiction (*r* = 0.503, *p* < 0.01).

Table 3 also reveals a strong correlation between media use during the lockdown and before the pandemic (*r* = 0.621, *p* < 0.01). Media use was positively correlated with both ADHD and smartphone addiction, but the correlation was significant only for media use *before* the pandemic, (for ADHD *r* = 0.162, *p* < 0.01; for smartphone addiction *r* = 0.118, *p* < 0.05).

### 3.4. Regression and moderated mediation analyses

We first tested a simple main effects regression model in which hope, smartphone use, and ADHD were predictors of general wellbeing. As a set, these three variables significantly predicted general wellbeing *F*_(3,329)_ = 45.83, *p* < 0.001. About 30% of the wellbeing variance was accounted for (*R*^2^ = 0.295). Furthermore, each of these predictors was *uniquely* associated with wellbeing (see Table 4). Hope yielded a large positive unique association with wellbeing (β = 0.427, *p* < 0.001). Adult ADHD (β = −0.135, *p* < 0.05) and smartphone addiction (β= −0.133, *p* < 0.05) had small negative unique associations with wellbeing. Thus, dispositional hope served as a main effect type of protective factor.

**Table 4:** Regression coefficients of hope, adult ADHD and smartphone addiction on wellbeing.

**Variable**	***B***	**SE**	***t***	***p*-Value**	**95% CI**
Constant	99.95	0.543	184.1	<0.001	98.88 to 101.01
Adult hope total	0.537	0.061	8.82	<0.001	0.417 to 0.657
Adult ADHD	−0.145	0.058	−2.39	<0.05	−0.259 to −0.030
Smartphone addiction	−0.124	0.050	−2.45	<0.05	−0.223 to −0.025

N = 333.

**Figure 1:**
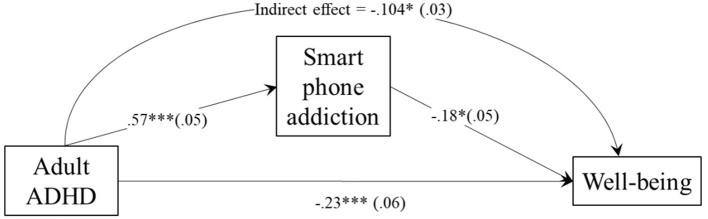
Mediation model of the ADHD effect on wellbeing through smartphone addiction. *p < 0.05; **p < 0.01; ***p < 0.001.

We then tested a mediation model in which smartphone use mediated the effect of ADHD on wellbeing. The mediation analysis yielded a significant mediation (indirect) effect [*b* = −0.104, 95% percentile CI (−0.170; −0.038)]. The direct effect of ADHD on wellbeing was also significant (*b* = −0.23, *p* < 0.001), showing partial mediation. Figure 1 illustrates these effects.

**Figure 2:**
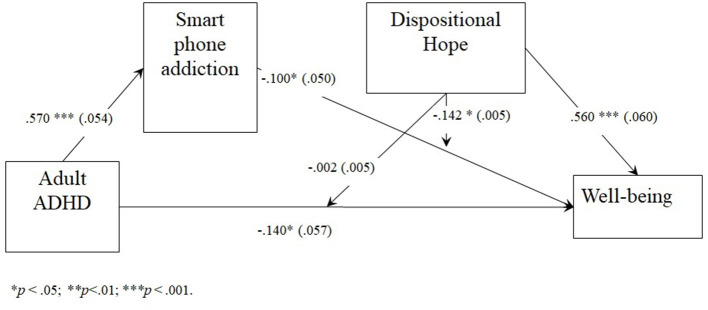
Moderated mediation model of the ADHD effect on wellbeing through smartphone addiction, moderated by dispositional hope. *p < 0.05; **p < 0.01; ***p < 0.001.

We then tested our moderated mediation model using Hayes PROCESS Macro (Model 15). The index for moderated mediation was significant, *b* = −0.008, 95% percentile CI (−0.0134; −0.0011), which did not contain zero, providing evidence for moderated mediation (Hayes, 2017). The model explains 32% of the variance of wellbeing (*R*^2^ = 0.317). The results, shown in Table 5 and Figure 2, revealed that dispositional hope moderated the effect of smartphone addiction on wellbeing. However, there was no significant moderation by dispositional hope on the effect between ADHD and wellbeing, *b* = −0.02, *p* > 0.05 Δ*R*^2^ = 0.0005. The full regression results can be seen in Table 5.

**Table 5:** Regression results for the a-path from adult ADHD to smartphone addiction, path b from smartphone addiction to wellbeing, and path c' from adult ADHD to wellbeing.

**Variable**	***b***	***SE***	***p*-Value**
**Model 1 a-path**			
Adult ADHD	0.57	0.054	<0.001
**Model 2 b/c' path**			
Adult ADHD	−0.14	0.057	<0.05
Smartphome addiction	−0.10	0.050	<0.05
Adult hope	0.56	0.06	<0.001
Adult ADHD × Adult hope	−0.002	0.005	>0.05
Smartphone addiction × Adult hope	−0.142	0.005	<0.05

N = 333.

Model for the a-path R^2^ = 0.25, F_(1,331)_ = 111.9, p < 0.001, Model for b-path and c'-path R^2^ = 0.317, F_(5,327)_ = 30.46, p < 0.001.

**Figure 3:**
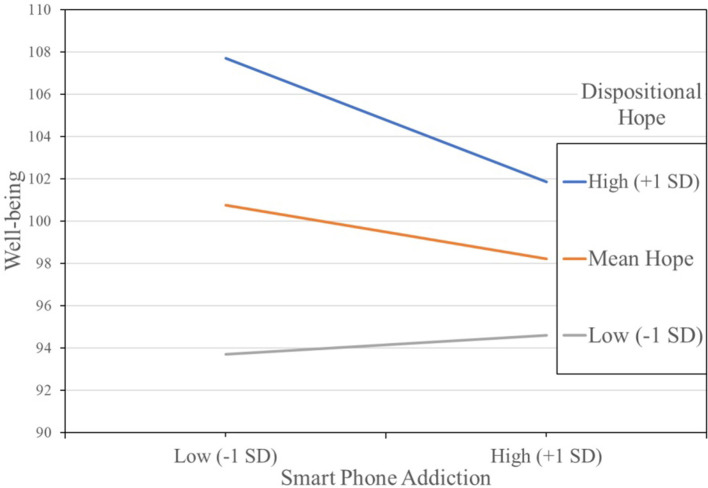
Effect of hope in the association between smartphone addiction and wellbeing.

Although hope did significantly moderate the smartphone effect on wellbeing (i.e., their interaction was significant), the form was different from what we expected. As shown in Figure 3, the harmful effect of smartphone addiction on wellbeing was the largest for those with high scores on dispositional hope. That is, the decline in wellbeing associated with high smartphone addiction was greatest for those with high dispositional hope (+1 *SD*), *b* = −0.133, 95% percentile CI (−0.208; −0.051). The harmful effect of smartphone addiction on wellbeing was weaker but also significant for those who scored at the average level of dispositional hope, *b* = −0.057, 95% percentile CI (−0.116; −0.002). The slope linking smartphone addiction to wellbeing was not significantly different from zero for those who had the lowest dispositional hope, *b* = 0.018, 95% percentile CI (−0.074; 0.091). In other words, dispositional hope was not a protective factor in the traditional sense of making a known risk factor (i.e., smartphone addiction) less impactful on the outcome (wellbeing).

Nonetheless, dispositional hope *was* a protective factor in that its main effect on wellbeing was so large that, even when paired with high smartphone addiction, the estimated mean on wellbeing was higher than any of the four low or medium dispositional hope estimated means in Figure 3. In sum, the combined main and moderation effects of dispositional hope on wellbeing during the COVID-19 pandemic were quite positive.

## 4. Discussion

The present study found most of the expected relationships among the study variables, thereby depicting harmful effects wellbeingof ADHD symptoms and smartphone addiction on wellbeing. At the same time, the results confirmed that dispositional hope served as a protective factor but only as the main effect (positive association with wellbeing) that essentially overrode the heightened harmful effect of smartphone addiction on those with high dispositional hope.

The study also found that self-reported time using internet-connected devices increased during the lockdown. Furthermore, the positive relationship between media use and adult ADHD found in other studies was replicated (*r* = 0.162, *p* < 0.05). Relatedly, we also found that overall time online was positively associated with smartphone addiction (*r* = 0.118, *p* < 0.01). The results are consistent with prior work suggesting that overall amount of electronic media consumption is associated with attention problems (e.g., Swing and Anderson, 2014).

Our results also showed that male students felt physically better during the pandemic than did female students, consistent with prior research (Magson et al., 2021; Sürücü et al., 2021). Contrary to our expectations, we did not find any significant differences related to gender for smartphone addiction and ADHD symptoms. However, we found a high value for smartphone use by Romanian college students, *M* = 30.6 (*SD* = 12.57). This mean was higher than the value of 25.26 (SD 10.78) reported by Kwon et al. (2013) for the general population. Being close to the cutoff points of 31 for boys and 33 for girls (Kwon et al., 2013), we can conclude that during the pandemic many Romanian students developed an addictive behavior to their smartphones.

Related to smartphone addiction, our correlation analysis revealed a moderate negative correlation with wellbeing (*r* = −0.304, *p* < 0.01) and a strong positive relation with adult ADHD symptoms (*r* = 0.503, *p* < 0.01). These zero-order associations were confirmed through the multiple regression analysis. Comparable results have been reported by other researchers studying the effects of excessive or problematic use of electronic devices—as in addictive behavior leading to poor wellbeing (Vanden Abeele et al., 2022) and mental health problems (Girela-Serrano et al., 2022). In particular, these results are consistent with the ones of Kwon et al. (2018, 2022) who found that smartphone addiction significantly influenced ADHD symptoms in university students. Other recent studies of the pandemic also showed that smartphone addiction is negatively related to wellbeing (Topan and Kuzlu Ayyildiz, 2021; Nayak and Pai, 2022) and hope (Çevik et al., 2020). Current studies on students' behavior during the COVID-19 pandemic period found an increase in addictive behavior related to alcohol consumption among college students associated with a decline in psychological health (Evans et al., 2021; Ryerson, 2022). Conversely, hopefulness proved to be positively related to wellbeing in difficult times (Evans et al., 2021; Putrawan et al., 2021; Ryerson, 2022).

Note, however, that there are studies that show that the lockdown due to the pandemic did not always have a significant harmful impact on mental health. In a meta-analysis of longitudinal studies and natural experiments on the psychological impact of the COVID-19 pandemic, Prati and Mancini (2021) concluded that the psychological impact of COVID-19 lockdowns was small in magnitude and highly heterogeneous, suggesting that lockdowns did not have uniform detrimental effects on mental health and that most people were psychologically resilient to their effects.

Perhaps our most important findings were as follows: (a) smartphone addiction appeared especially harmful for the wellbeing of those with high dispositional hope and (b) the beneficial effects of dispositional hope appeared to counteract the effect of smartphone addiction on wellbeing. It is interesting to note that, even among those students who reported high smartphone addition, those with high dispositional hope reported greater levels of wellbeing than those with low levels of hope *regardless of smartphone addition*. In other research, hope has been shown to moderate the relationship between overall wellbeing and negative life events (Hirsch et al., 2012; Christens et al., 2013; Visser et al., 2013; Sun et al., 2014; Hellman and Gwinn, 2017; Munoz et al., 2020; Li et al., 2021; Sparks et al., 2021).

Our research question was whether hopefulness still had the same effect in the new challenges related to lockdowns due to the COVID-19 pandemic. The moderated mediation analysis in our research showed that dispositional hope played a protective role in students' wellbeing, just not in the way that we had expected. The study also replicated the well-established relationship between attention deficit symptoms and problematic behavior—addiction to smartphones—and wellbeing among Romanian college students. Interestingly, there was no significant moderation of hope in the relation between attention problems and wellbeing in our participants.

### 4.1. Limitations and future directions

The most obvious limitation (see text footnote 2) is that cross-sectional studies do not provide strong tests of causal direction. Thus, our findings should be interpreted cautiously.

Another limitation is the relatively small number of men in the sample. This limits the generalizability of the findings somewhat. However, it is important to note that (to our knowledge) there is no theoretical or empirical reason to expect that the associations of the key variables, i.e., their slopes, should differ between women and men. There are good reasons to expect some gender main effects whenever one is studying media effects and wellbeing, but not slope differences. Another sample limitation is that all participants were social sciences and humanities students.

In contrast to the limitations, among the strengths of this particular data set is that it used a vastly understudied population, namely, college students in Romania. That the study replicated several common findings from primarily Western samples strengthens the notion that the findings regarding hope, media use, media addiction, ADHD, and wellbeing replicate across a wide range of cultures.

Additional research is needed to address the causal direction of the discovered associations. Experimental studies are best suited to this task, but large-scale intervention studies are very expensive and time-consuming. Longitudinal studies would also be helpful in testing which variable changes precede vs. follow other variable changes.

An additional aspect that needs further clarification is the relationship between media use, smartphone addiction, and attention problems. Numerous cross-sectional, and a few longitudinal and experimental studies now suggest that high exposure to some types of electronic media may actually harm basic attention skills and thereby lead to significant behavior problems.

## 5. Conclusion

In conclusion, our findings confirmed that during lockdown there were significant negative associations between attention problems, smartphone addiction, and wellbeing and that dispositional hope may be an important protective factor. The findings also suggest that developing and instituting programs that reduce smartphone addiction and increase dispositional hope could be effective ways to improve the wellbeing of a substantial portion of students.

## Data availability statement

The raw data supporting the conclusions of this article will be made available by the authors, without undue reservation.

## Ethics statement

The present study received the approval of the Ethics Committee (no. 13003/0-1/04.03.2020, RCE 2020-28) of the West University of Timişoara. The patients/participants provided their written informed consent to participate in this study.

## Author contributions

RT and CA contributed to conception and design of the study. AF, BA-G, and AC collected the data and organized the database. RT and MM performed the statistical analysis. RT wrote the first draft of the manuscript. CA, KB, MM, AF, BA-G, and AC wrote sections of the manuscript. All authors contributed to manuscript revision, read, and approved the submitted version.

## Footnotes

^1^This study focuses on ADHD symptoms using a well-validated self-report continuous measure. It does not have a dichotomous clinical diagnosis of ADHD as a variable.

^2^We acknowledge that testing mediation models with cross-sectional data is not the optimal way to test direction of causality. Experimental and longitudinal designs are needed to further test our hypotheses. Nonetheless, our theoretical model does predict what the cross-sectional data should look like, and therefore the study provides a good opportunity for the model to be disconfirmed.
